# VR-based automated suturing skill assessment in pediatric robotic surgery

**DOI:** 10.1007/s11548-026-03659-3

**Published:** 2026-04-30

**Authors:** Saul Alexis Heredia Perez, Enduo Zhao, Murilo Marques Marinho, Kyoichi Deie, Mamoru Mitsuishi, Kanako Harada

**Affiliations:** 1 School of Medicine, The University of Tokyo, Hongo 7-3-1, Bunkyo City, Tokyo 113-8654 Japan; 2 School of Engineering, The University of Tokyo, Hongo 7-3-1, Bunkyo City, Tokyo 113-8654 Japan

**Keywords:** Surgical skills assessment, Virtual reality simulation, Pediatric surgery

## Abstract

**Purpose:**

Objective assessment of robotic surgical skills is particularly important in pediatric surgery, where limited case volume restricts training opportunities. This study presents a virtual reality (VR)-based framework for automated evaluation of robotic suturing skills in a neonatal surgical scenario and investigates its agreement with expert video-based assessment.

**Methods:**

A real-time VR simulator was developed to emulate neonatal robotic suturing with the SmartArm system. An automated skills assessment module was implemented using an 11-point subset of a validated 29-point suturing checklist. Each checklist item was reformulated into quantitative geometric and kinematic criteria directly extracted from the simulation . Ten suturing trials were recorded and independently evaluated by an expert pediatric surgeon using video review. Automated scores were compared with expert scores using accuracy, precision, recall, and F1-score.

**Results:**

The simulator enabled stable real-time execution of robotic suturing tasks and deterministic extraction of performance metrics. The automated assessment achieved an accuracy of 67.3%, with a precision of 0.933, recall of 0.560, and F1-score of 0.700 relative to expert evaluation. Higher agreement was observed for clearly defined metrics, while discrepancies were primarily associated with criteria dependent on visual judgment in 2D video assessment.

**Conclusion:**

VR-based automated assessment of robotic pediatric suturing is feasible and provides objective, repeatable evaluation of performance. By translating clinically defined checklist items into measurable simulation-derived parameters, the proposed framework offers a scalable alternative to manual video-based skills assessment in robotic surgery training.

## Introduction

Esophageal atresia is a rare congenital condition requiring complex surgical repair shortly after birth. Due to the low incidence of these cases and the technical difficulty of neonatal suturing in a confined workspace, opportunities for pediatric surgeons to acquire and maintain advanced minimally invasive and robotic skills in the operating room are limited. Robot-assisted surgery can improve dexterity and visualization in minimally invasive procedures; however, its adoption in pediatric surgery, particularly in neonates, remains limited. Only a small number of robot-assisted thoracoscopic repairs of esophageal atresia in neonates have been reported [1], further constraining hands-on training opportunities and motivating the need for simulation-based training and assessment methods.

**Fig. 1:**
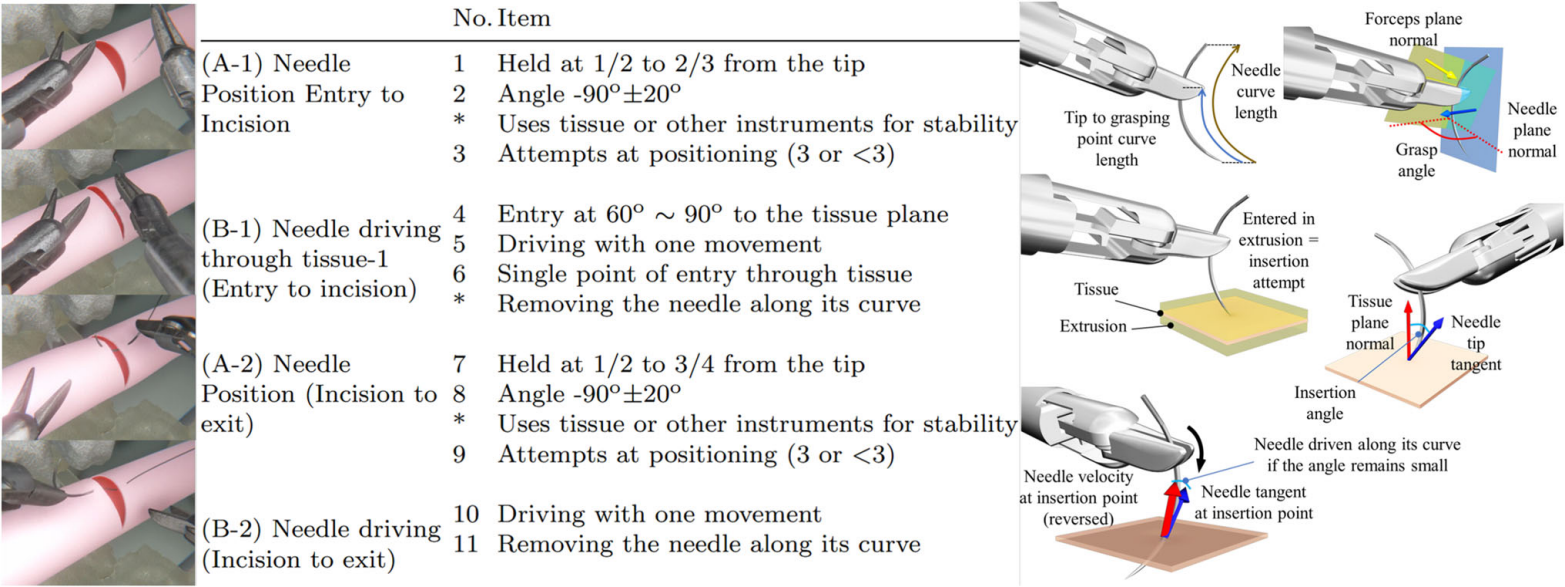
Overview of the VR-based automated suturing skills assessment. Left: VR simulator snapshots illustrating key suturing steps. Center: corresponding checklist items used for skills evaluation. Right: geometric and kinematic parameters extracted from the simulation for automated scoring

Virtual reality (VR) simulation provides a controllable and repeatable environment for surgical training, with direct access to geometric and kinematic information that enables automated performance assessment. While prior work has demonstrated VR-based skills evaluation for manual suturing and adult robotic surgery [2, 3], automated VR-based skills assessment tailored to robotic pediatric surgery remains unexplored. In this work, we present a VR-based framework that reformulates an established suturing checklist into measurable simulation-based criteria and evaluates the feasibility of automated assessment compared to expert video-based evaluation [4].

The proposed approach extends previous developments on the SmartArm robotic platform [5], which focused on teleoperation feasibility and safety in neonatal surgery but did not address objective performance evaluation. Moreover, although the neonatal chest model by Deie and colleagues [6] enabled manual video-based assessment, it relied on subjective expert interpretation. By integrating this anatomical model into a VR environment with deterministic parameter extraction, the present work enables repeatable and observer-independent automated scoring specifically designed for robotic pediatric suturing.

## Methods

A VR simulator was developed to reproduce a robotic neonatal suturing scenario using the SmartArm robotic system [5]. The simulator provides real-time access to pose, contact states, and kinematic information of robotic tools, needle, and tissue, which are used for automated skills assessment. The simulated anatomy was based on a neonatal chest model [6], including the ribcage, heart, aorta, and a synthetic esophagus with a semicircular incision representing esophageal atresia. Only the esophageal region surrounding the incision was modeled as deformable, while the remaining anatomy was treated as static geometry (See Fig. 1—left). The simulator was implemented using the PhysX physics engine (NVIDIA Corporation, USA).

**Fig. 2:**
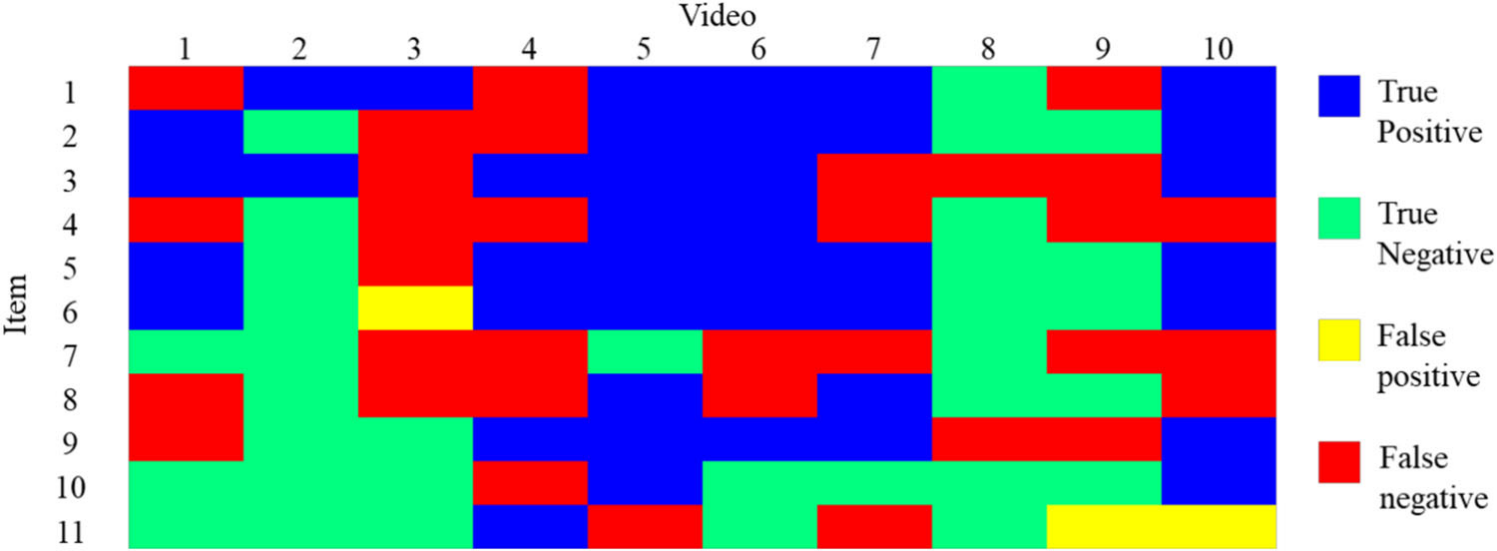
Confusion matrix for each item from the 11-point checklist for each evaluated video

Automated surgical skills assessment was implemented within the VR simulator based on the 29-point suturing checklist proposed by Moorthy and colleagues [4]. To evaluate feasibility, this study focused on the needle positioning and driving phases, corresponding to the first 14 checklist items. Three items marked with an asterisk (*) were not included in this study, as they do not apply in pediatric surgery. The item “Uses tissue or other instruments for stability” was omitted because, in pediatric surgery, unnecessary tool–tissue contact is minimized to prevent tissue damage. The item “Removing the needle along its curve” was also excluded. In this task, the needle was passed through both tissue edges in a single continuous motion and withdrawn without exiting at the first edge before proceeding to the second (see Fig. 1—left). Because this technique differs from the anastomotic approach assumed in the original checklist, the item was not applicable to the present evaluation criteria. This yielded an 11-point subset used for automated evaluation (see Fig. 1—center). Each checklist item was scored in a binary manner (0 or 1). Since the original checklist was designed for manual video-based assessment, its qualitative descriptions were reformulated into quantitative criteria based on geometric, kinematic, and temporal parameters directly obtainable from the simulation.

Specifically, the checklist items were mapped to measurable features such as needle grasp position and orientation, number of insertion attempts, insertion angle relative to the tissue surface, continuity of needle motion, and alignment between needle velocity and curvature during driving (see Fig. 1—right). These parameters were computed deterministically from the simulated state without user intervention, enabling objective and repeatable assessment of suturing performance. The resulting automated scores were compared against expert video-based evaluations to assess feasibility.

## Experiments and results

The proposed automated skills assessment was evaluated by comparison with video-based expert assessment [6]. Ten VR-simulated suturing trials focusing on needle positioning and driving were recorded, including five representative good and five intentionally poor performances. An expert pediatric surgeon independently scored the videos using the 11-point checklist defined in this study, and these scores were treated as ground truth. Figure 2 shows the confusion matrix. The VR simulator enabled real-time execution of the robotic suturing task under conditions representative of neonatal surgery. Comparison between automated and expert scores yielded an accuracy of 67.3%, a precision of 0.933, a recall of 0.560, and an F1-score of 0.700, indicating substantial agreement and conservative scoring behavior of the automated assessment.

## Discussion and conclusion

This work demonstrates the feasibility of VR-based automated assessment for robotic pediatric suturing by reformulating a clinically established checklist into measurable simulation-based criteria. The automated method reproduced expert scores with an accuracy of 67.3% and a low false-positive rate, indicating conservative and consistent scoring behavior. Higher agreement was observed for clearly defined metrics, while lower agreement occurred for metrics that are difficult to judge from 2D endoscopic video, such as needle insertion angle. In these cases, direct geometric computation within the simulator may provide more objective evaluation than manual video-based assessment. Overall, the results suggest that VR-based automated skills assessment can reduce subjectivity, enable repeatable evaluation, and support real-time feedback, making it a promising complement to traditional assessment methods for pediatric robotic surgery training. Future work will extend the proposed framework to additional phases of the suturing task, particularly knot tying, by incorporating the remaining checklist items and defining corresponding measurable criteria. This will enable a more comprehensive evaluation of surgical performance within the VR environment.
